# Effectively Screening for Coronary Artery Disease in Patients Undergoing Orthotopic Liver Transplant Evaluation

**DOI:** 10.1155/2016/7187206

**Published:** 2016-06-22

**Authors:** Bryan C. Lee, Feng Li, Adam J. Hanje, Khalid Mumtaz, Konstantinos D. Boudoulas, Scott M. Lilly

**Affiliations:** ^1^Department of Internal Medicine, The Ohio State University Wexner Medical Center, Columbus, OH 43210, USA; ^2^Division of Gastroenterology, Hepatology and Nutrition, The Ohio State University Wexner Medical Center, Columbus, OH 43210, USA; ^3^Division of Cardiovascular Medicine, The Ohio State University Wexner Medical Center, Columbus, OH 43210, USA

## Abstract

Coronary artery disease (CAD) is prevalent in patients with end-stage liver disease and associated with poor outcomes when undergoing orthotopic liver transplantation (OLT); however, noninvasive screening for CAD in this population is less sensitive. In an attempt to identify redundancy, we reviewed our experience among patients undergoing CAD screening as part of their OLT evaluation between May 2009 and February 2014. Demographic, clinical, and procedural characteristics were analyzed. Of the total number of screened patients (*n* = 132), initial screening was more common via stress testing (*n* = 100; 75.8%) than coronary angiography (*n* = 32; 24.2%). Most with initial stress testing underwent angiography (*n* = 52; 39.4%). Among those undergoing angiography, CAD was common (*n* = 31; 23.5%). Across the entire cohort the number of traditional risk factors was linearly associated with CAD, and those with two or more risk factors were found to have CAD by angiography 50% of the time (OR 1.92; CI 1.07–3.44, *p* = 0.026). Our data supports that CAD is prevalent among pre-OLT patients, especially among those with 2 or more risk factors. Moreover, we identified a lack of uniformity in practice and the need for evidence-based and standardized screening protocols.

## 1. Introduction

The average age of patients undergoing orthotopic liver transplant (OLT) continues to increase, as do higher model for end-stage liver disease (MELD) scores and an increasing number of comorbidities for cardiovascular disease, particularly obesity and metabolic syndrome [1–3]. Coronary artery disease (CAD) is prevalent within the OLT population and is associated with greater short and intermediate morbidity and mortality [4, 5]. Moderate to severe coronary artery disease (≥50% stenosis) in pre-OLT patients has been reported to be anywhere from 2.5% to 27% [6–8]. Carey et al. also found 13.3% to have significant CAD (40%–70% stenosis) in those without history of angina, myocardial infarction, percutaneous transluminal coronary angioplasty, or coronary artery bypass grafting. As many patients who have obstructive CAD are asymptomatic, pre-OLT risk assessment may be obscured by the clinical history [6]. Moreover, previous studies have not ascribed to uniform definitions of significant CAD and an accurate prevalence is therefore difficult to ascertain. Existing stress testing modalities have limitations when employed in the end-stage liver disease (ESLD) population. Single-photon emission computed tomography (SPECT) has been associated with a low sensitivity (62%) for coronary disease, perhaps referable to the ineffectiveness of adenosine or regadenoson to achieve adequate microvascular vasodilation [9]. A recent systematic review revealed the shortcomings of dobutamine stress echocardiography (DSE) in this population, having a sensitivity of 32% for detecting CAD [10]. Current guidelines from the American Association for Study of Liver Diseases continue to recommend DSE for the screening of CAD with coronary angiography used to confirm a positive test [11]. In contrast, the American College of Cardiology (ACC) and the American Heart Association (AHA) suggest using noninvasive testing to screen for CAD based on the number of risk factors, with 3 or more being the most reasonable [12]. The expanded use of coronary angiography as screening or confirmatory modality may be associated with increased bleeding or vascular complications, although this is not a uniform observation [13–17]. The goal of the present report is to describe our experience with CAD screening in the pre-OLT population.

## 2. Methods

This was a retrospective review, which was approved by the Ohio State University Medical Center's Institutional Review Committee using data from the electronic medical record. The inclusion criteria were all patients ≥18 years of age seen at the OSU Division of Hepatology Clinic and evaluated for liver transplant between May 1, 2009, and February 1, 2014. Deidentified data from charts were used to construct a database of liver transplant candidates with information on risk factors and preoperative testing. All data regarding complications from left heart catheterization were extracted from an existing database that tracks all patients undergoing diagnostic and interventional procedures. Pre- and postcoronary angiography events were identified using National Cardiovascular Data Registry (NCDR) definitions and included cardiogenic shock, heart failure, cerebrovascular accident/stroke, tamponade, new requirement for dialysis, vascular complications, blood transfusion, hematoma, bleeding at access site, or retroperitoneal bleed [18]. Traditional risk factors for CAD were used and included age (men > 45, female > 55), hypertension, hyperlipidemia, diabetes mellitus, obesity (BMI > 30), and family history of premature CAD (diagnosis of CAD in a first-degree male relative before 55 or first-degree female relative before 65) [19]. Coronary artery disease was defined as at least 1 vessel with a ≥50% stenosis; obstructive disease was defined as ≥70% stenosis on coronary angiography. Primary angiography represents those who bypassed noninvasive CAD evaluation due to their risk factor profile. Secondary angiography was defined as those with initial noninvasive CAD testing however underwent coronary angiography afterwards.

Categorical variables were compared using the chi-square test. For continuous variables, between group differences were analyzed with univariate analysis of variance (ANOVA). All *p* values presented are 2-tailed; ≤0.05 was considered statistically significant. Analyses were performed with Stata 12.0 software (Stata Corp., College Station, TX).

## 3. Results

**Figure 1:**
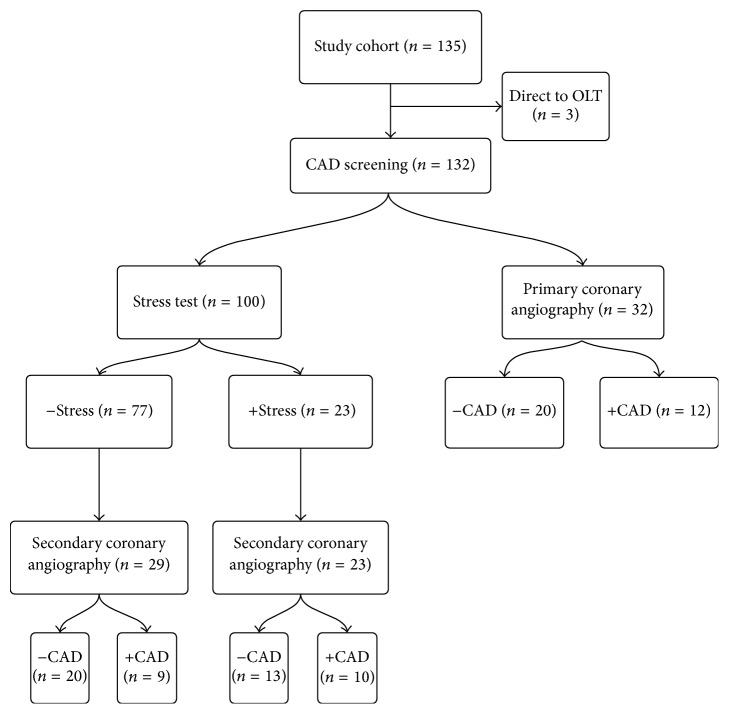
Positive stress test is defined as signs of ischemia on ECG during exercise, signs of ischemia on nuclear perfusion stress, and/or signs of infarction on perfusion study. Positive coronary artery disease was defined as at least a single vessel with ≥50% stenosis. ECG: electrocardiogram.

Within the specified period, a total of 135 patients underwent evaluation for OLT and 132 subsequently underwent CAD screening. Thirty-two (24.2%) of these patients underwent primary coronary angiography while 100 (75.8%) had stress test performed initially. Of the patients who were initially stressed, 52 eventually underwent coronary angiography (Figure 1).

The mean age of the cohort was 56 (range 32–71) and more commonly male (69.7%). The most common cause of liver disease was hepatitis C (56, 42.4%). Cardiac risk factors were prevalent, with 41.2% having ≥2 traditional risk factors (Table 1). Within the cohort 84 (63.3%) underwent coronary angiography, 32 with no prior stress test, 23 with a positive stress test, and 77 with a negative stress test (Figure 1).

For coronary angiography the radial access approach was primarily utilized (60.7%). Although the catheterization laboratory employs a radial-first approach, femoral access was utilized in nearly 40% of the cases. These cases reflect occasional anatomical limitations of radial angiography, as well as operator bias. There were no intra- or postprocedure events based upon NCDR definition [18]. Eleven (13.1%) patients were readmitted to the hospital within 30 days, 5 of these were due to intra-abdominal infection, 2 were due to decompensated cirrhosis, and the rest were due to TACE, hyponatremia, upper GI bleed, and viral gastroenteritis (Table 2).

**Figure 2:**
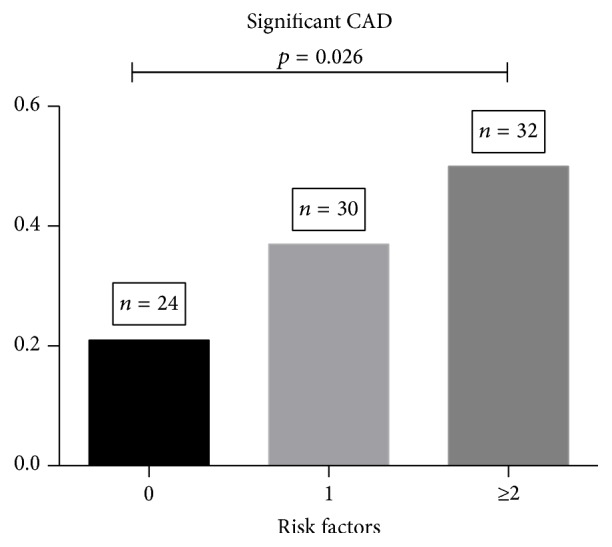
Incidence of CAD based upon number of risk factors that include age, hyperlipidemia, hypertension, family history of premature heart disease, diabetes, and obesity. CAD: coronary artery disease.

Coronary artery disease (≥50% stenosis) was identified in 31 (23.5%) patients. Of those with CAD, 7 (5.3%) demonstrated obstructive disease (≥70%). On univariate analysis no single risk factor was associated with finding CAD. Although recent data has identified poorer cardiovascular outcomes peri-OLT among those with higher MELD scores, we did not find a significant relationship between higher MELD (>15) and the presence of CAD in this cohort (OR 1.17, HR 0.29–4.64, *p* = 0.83) [20]. Moreover, the general relationship between CAD risk factors and the presence of angiographic CAD was not significantly affected when the cohort was stratified by MELD greater than or less than 15. The number of traditional risk factors was linearly associated with angiographic coronary artery disease, and those with 2 or more risk factors were found to have CAD by angiography 50% of the time (OR 1.92; CI 1.07–3.44, *p* = 0.026, Figure 2).

## 4. Discussion

Limitations of noninvasive testing for CAD in the pre-OLT population have led some to propose a wider adoption of pretransplant coronary angiography. However, there are no existing guidelines based on contemporary data that address the role of coronary angiography in this setting. Based on existing observational data [21], our institution recently employed lower thresholds for pretransplant angiography. This early experience has demonstrated that coronary angiography is safe and might be considered in all patients undergoing pre-OLT CAD screening, particularly those with 2 or more traditional risk factors for CAD.

Complications of cirrhosis include coagulopathy and renal hypoperfusion, increasing the risk of bleeding and associated nephropathy [22]. Despite these presumed risks, we observed no adverse events in the 132 cases reported herein. A “radial-first” approach and the use of strict contrast thresholds may have minimized the expected risk. This is consistent with recent studies and continues to support the safety of coronary angiography in this population [14, 15, 23].

A favorable safety profile may permit wider spread adoption of angiography, but it remains unclear which patients should bypass noninvasive screening and proceed directly to the catheterization laboratory. A major residual issue is determining which preprocedural characteristics predict the presence of CAD in those with ESLD. In the present report, no single CAD risk factor correlated with the presence of CAD. However, among those with 2 or more risk factors the incidence of CAD was 50%. Others have formerly similar results: 2 or more risk factors increased the likelihood of having moderate to severe CAD in any vessel [8].

Although coronary angiography is safe and improves the detection of CAD in pre-OLT patients, whether or not this leads to a reduction in peritransplant cardiovascular morbidity and mortality is unresolved but critically important. One multicenter review found that pre-OLT patients with angiographically proven obstructive CAD treated with current CAD strategies had no significant difference in survival after OLT compared to those without significant CAD [24]. This was in stark contrast to an earlier study showing a 50% mortality 1 to 3 years after OLT; of note, none received coronary stenting in that cohort [5]. More recently a single-center study has shown a benefit in OLT outcomes after increasing their reliance on coronary angiography over time [25]. The greatest reduction in mortality occurred when patients were referred to angiography based on the presence of certain cardiac risk factors (age, smoking, family history of CAD, diabetes, hypertension, prior CAD, and obesity).

Our study does include limitations. The data comes from a large tertiary referral center and may not be representative of the burden of CAD, CAD risk factors, or the spectrum of liver disease in other centers. Ascertainment bias is a possibility. Complications from procedures would only have been captured if they occurred during the hospitalization and were readmitted here or to our affiliated hospital within 30 days. That being said, these patients were undergoing transplant evaluation here along with the bulk of their primary and consultative care. Moreover, this is a retrospective review and has the potential for accompanying bias and confounding. Additionally, this observational study took place at a time when the prevalence of CAD in OLT patients was being increasingly recognized, as were the limitations of noninvasive testing. Accordingly, the effect of those realizations on practice patterns cannot be ascertained. Lastly, due to the low volume of liver transplants performed over the time span of the study we cannot comment on the impact angiography-associated treatment or the denial of OLT based on screening had on outcomes. That being said, the results reported herein are from a comparably large population with respect to other studies of CAD in the pre-OLT patients, and many of our findings are in agreement with published literature. Taken together, we submit that the present results, together with others, support pretransplant coronary angiography at least in all pre-OLT patients with 2 or more traditional CAD risk factors [26]. Whether or not wider spread adoption will lead to improving outcomes awaits ongoing prospective reports.

In our institutional experience coronary angiography should be performed in patients undergoing CAD screening for OLT who have 2 or more traditional cardiac risk factors. By using this guideline the hope is to uncover CAD that can be missed by noninvasive testing and improve survival after OLT. As we will undoubtedly find more CAD in the pre-OLT population, continuing to track outcomes will remain important specifically in regard to PCI thresholds.

## Figures and Tables

**Table 1:** Clinical characteristics of our entire study cohort.

Age, years	56 ± 7.72
Male, *n* (%)	92 (69.7)
Caucasian, *n* (%)	117 (88.6)
African American, *n* (%)	8 (6.1)
Hepatitis C cirrhosis, *n* (%)	56 (42.4)
Nonalcoholic steatohepatitis, *n* (%)	33 (25)
Alcohol cirrhosis, *n* (%)	21 (15.9)
Model of end-stage liver disease	16.1 ± 5.94
Hypertension, *n* (%)	49 (37.1)
Diabetes mellitus, *n* (%)	45 (34.1)
Tobacco abuse, *n* (%)	47 (35.6)
Hyperlipidemia, *n* (%)	18 (13.6)
Obese, *n* (%)	36 (27.3)
Family history of premature heart disease, *n* (%)	28 (21.2)
Alcohol abuse, *n* (%)	27 (20.4)
No coronary risk factors, *n* (%)	37 (27.2)
One coronary risk factor, *n* (%)	43 (31.6)
Two or more coronary risk factors, *n* (%)	56 (41.2)
Serum creatinine	1.04 ± 0.62
INR	1.57 ± 0.44
Platelet count	83.85 ± 43.16

Values are shown as mean ± standard deviation.

**Table 2:** Characteristics of coronary angiography.

Total coronary angiography, *n* (%)	84 (63.6%)
Radial approach, *n* (%)	51 (60.7%)
Femoral approach, *n* (%)	33 (39.3%)
Coronary artery disease with ≥50% stenosis, *n* (%)	31 (36.9%)
Coronary artery disease with ≥70% stenosis, *n* (%)	7 (8.3%)
Major adverse events, *n* (%)	0
Minor adverse events, *n* (%)	0
Readmission within 30 days, *n* (%)	11 (13.1%)

Total coronary angiography percentage is of total cohort studied, *n* = 132.
